# Protocol for cytoskeleton staining of the semi-adherent multiple myeloma cell line RPMI 8226 by immunofluorescence

**DOI:** 10.1016/j.xpro.2024.103060

**Published:** 2024-05-02

**Authors:** Vasty Osei-Amponsa, Valentin Magidson, Kylie J. Walters

**Affiliations:** 1 Protein Processing Section, Center for Structural Biology, National Cancer Institute, National Institutes of Health, Frederick, MD 21702, USA; 2 Optical Microscopy and Image Analysis Laboratory, Center for Cancer Research, National Cancer Institute, National Institutes of Health, Frederick, MD 21702, USA

**Keywords:** Cell Biology, Cell culture, High-Throughput Screening, Immunology, Microbiology, Antibody

## Abstract

Preservation of fine cellular details of semi-adherent or suspension cells for imaging by immunofluorescence is challenging. This protocol enables staining of floating cells with minimal morphological distortions, as we demonstrate with the semi-adherent multiple myeloma cell line RPMI 8226. We describe steps to better preserve structural details by fixing, permeabilizing, and staining cells in solution, while minimizing the number of centrifugation steps and centrifugation g-force.

For complete details on the use and execution of this protocol, please refer to Osei-Amponsa et al.^1^

## Highlights

• Protocol for immunofluorescence microscopy of semi-adherent cells
• Steps for preparing multiple myeloma RPMI 8226 cells for immunostaining
• Approach for minimal distortion of cell morphology by reducing centrifugation

## Abstract (editor highlights)

Publisher’s note: Undertaking any experimental protocol requires adherence to local institutional guidelines for laboratory safety and ethics.

## Graphical abstract

**Figure:**
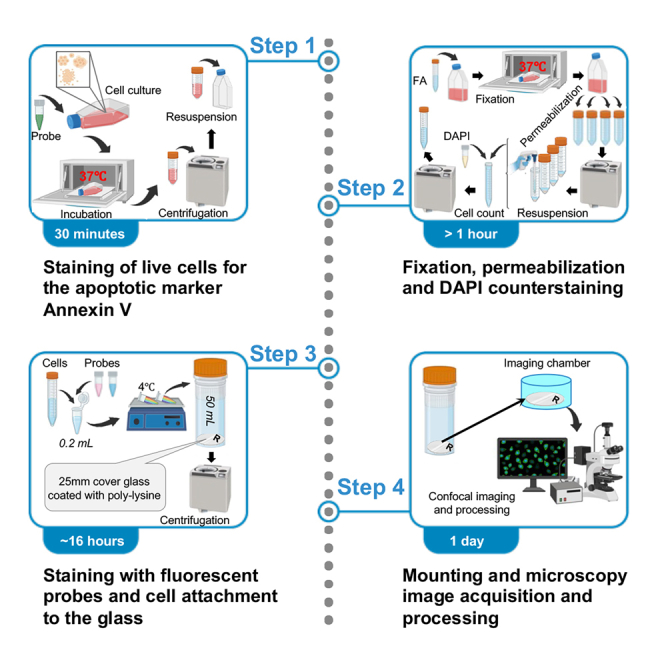

## Before you begin

Challenges of immunostaining cells from suspension cultures have been addressed by several protocols.^2^^,^^3^^,^^4^^,^^5^ Most approaches involve cytocentrifugation,^6^^,^^7^ whereby some degree of deformation is tolerated to promote strong attachment of suspension cells to a glass slide. Drying cells on a slide, with or without heating, is often involved to facilitate this attachment, after which suspension cells are processed similarly to adherent ones. We devised an alternative approach, optimized to preserve cell morphology, by attaching cells to glass only at the final step of the protocol and using only moderate centrifugal force. By using actin staining of RPMI 8226 cells, we were able to demonstrate that application of this protocol avoids cellular deformation, even for thin protrusions at the cell surface. This protocol can be fine-tuned for specific applications by adjusting the g-force of the last centrifugation step such that preservation of cell morphology is balanced against the strength of cellular attachment to the glass.

### Cell culture

**Timing: 2–4 days**

1. Culture semi-adherent cells of interest (e.g., RPMI 8226) in 20 mL of the appropriate media (e.g., RPMI media supplemented with 10% fetal bovine serum) in a T75 cell culture flask to desired density (e.g., ∼80% confluency).

***Note:*** Sufficient quantities of cells for the staining should be generated, accounting for loss during sample preparation.

### Fixing reagents preparation

**Timing: 30 min**

2. Prepare fixation reagents.

**Table:** Fixation reagent for 20 mL cell suspension

Reagents	Final concentration	Volume
37% Formaldehyde (FA)	4% after addition to cells	2.457 mL
10× Phosphate-Buffered Saline (PBS)	1× in fixation reagent	0.273 mL
TOTAL	N/A	2.73 mL

**CRITICAL:** Formaldehyde is toxic, handle in a fume hood.

### Permeabilization solution preparation

**Timing: 30 min**

3. Prepare 1 M Tris pH 8.0 solution.
4. Prepare 10% v/v stock of Triton X-100 in H_2_O.
5. Prepare permeabilization/quenching buffer.

**Table:** Permeabilization quenching buffer for 20 mL fixed cells

Reagents	Final concentration	Volume
1 M Tris pH 8.0	200 mM	32 mL
10% Triton X-100	0.1%	1.6 mL
H_2_0 (Milli Q)	N/A	126.4 mL
TOTAL	N/A	160 mL

***Note:*** Permeabilization/quenching buffer was made fresh prior to use. However, we do not expect any problems with long term storage of this buffer at 4°C.

### Fluorophore conjugation to antibody

**Timing: 1–2 h**

6. Conjugate antibodies of interest with the desired fluorophores.

***Note:*** For this protocol, we used the commercial Biotium Mix-n-Stain Kit. It is important to select probes with no spectral overlap. Biotium kits can work with both carrier-free and some non-carrier-free antibodies; see manufacturer protocol for details: https://biotium.com/wp-content/uploads/2017/10/PI-Mix-n-Stain-Antibody-Labeling-Kits.pdf.

***Alternatives:*** Other commercial kits are also available for antibody conjugation, and commercially available primary antibodies already conjugated to fluorophores can also be used.

### Cover glass coating

**Timing: 1 day**

7. Prepare a 0.01% poly-lysine solution in Milli Q water.
8. Rinse desired quantity of 25 mm round cover glasses with Milli Q water.
9. Wipe each cover glass dry with a lens cleaning tissue and lay it on parafilm.
10. Label the coating side of the glass with a fine tip Sharpie marker (e.g., write the letter ‘R’).
11. Pipette 1 mL of poly-lysine solution onto each cover glass with the labeled side facing upwards.
12. Incubate at 20°C–23°C for 4 h.
13. Rinse poly-lysine solution off the cover glasses under running Milli Q water.
14. Air-dry the cover glasses in a cell culture hood 12–15 h.

***Note:*** The coated and labeled cover glasses can be stored for at least a few weeks at 4°C in a closed container.

***Alternatives:*** This protocol uses lens cleaning tissues to wipe the cover glass. Kimwipes can also be used.

## Key resources table

**Table:** 

REAGENT or RESOURCE	SOURCE	IDENTIFIER
**Antibodies**		

Rabbit anti-TUBB4A (1:30 dilution)	Abcam	Cat#ab179509; RRID: AB_2716759

**Chemicals, peptides, and recombinant proteins**		

Tris (hydroxymethyl) aminomethane	Thermo Fisher Scientific	Cat#17926
Triton X-100	Roche	Cat#10789704001
Fetal bovine serum	R&D Systems	Cat#S12450
37% formaldehyde	Electron Microscopy Sciences	Cat#15686
DAPI nuclear stain	Thermo Fisher Scientific	Cat#D1306
Phalloidin ATTO-643 fluorescent dye (1:100 dilution)	ATTO-TEC	Cat#AD643-81
Poly-L-lysine	Sigma	Cat#P8920
Mounting medium	Biotium	Cat#23008
RPMI-1640 media	American Type Culture Collection	Cat#30-2001
10× phosphate-buffered saline (pH 7.4)	Thermo Fisher Scientific	Cat#70011044
Sodium azide	Mallinckrodt	Cat#1953-57 (Been discontinued)

**Critical commercial assays**		

Biotium Mix-n-Stain kit for CF568	Biotium	Cat#92255
Annexin V – CF488A conjugates	Biotium	Cat#29005

**Experimental models: Cell lines**		

Human: RPMI 8226 multiple myeloma cell line	American Type Culture Collection	Cat#CRM-CCL-155

**Software and algorithms**		

Andor Fusion software, including deconvolution	Oxford Instruments	N/A
Imaris	Oxford Instruments	N/A
ImageJ	Schneider et al.	N/A

**Other**		

Precision cover glasses	Thorlabs	Cat#CG15XH1
Parafilm	Sigma	Cat#sc-200311
Lens cleaning tissues	Thorlabs	MC-5
50 mL conical tubes	Falcon (Corning)	Cat#352098
0.5 mL Eppendorf tubes	Sigma	BR780536
50 mL flat bottom tubes	TESC Inc	Cat#CT-168000
15 mL conical tubes	Falcon (Corning)	Cat#352095
Rocking platform model 100	VWR	Model 100
Incu-Shaker mini	Benchmark	H1001-M
Avanti J-E centrifuge	Beckman Coulter	369001
Chamlide magnetic imaging chamber	Live Cell Instrument	Cat#CMB-25
Optic tweezers	Thorlabs	Cat# TZ1
Pasteur pipette, LDPE	ABDOS Life Sciences	Cat#P31205
Sharpie marker	Uline	S-19421BL
T75 flask	Corning	430641
Leica DMi8 microscope	Leica Microsystems	DMi8
Yokogawa CSU-W1 spinning disk	Yokogawa	SCW001
Andor Zyla 4.2 sCMOS camera	Oxford Instruments	Zyla 4.2P-USB3-W
63× NA1.4 oil lens	Leica Microsystems	11506350
63× NA1.2 water lens	Leica Microsystems	11506346

All resources reported are US sources.

## Step-by-step method details

### Staining of live cells for the apoptotic marker annexin V

**Timing: 30 min**

This part of the protocol describes steps for staining apoptotic cells prior to fixation. This step can be bypassed to perform immunofluorescence staining of fixed cells as described in step 2.

1. Apoptotic staining of semi-adherent cells.
  a. Pre-warm an orbital shaking incubator (e.g., Incu-Shaker mini) to 37°C.
  b. Add Annexin V conjugate solution to the cell culture media within the T75 flask.
    ***Note:*** In this protocol, we use 10 μg Annexin V to stain 20 mL of cell media. This step is performed following the manufacturer’s instructions: https://biotium.com/wp-content/uploads/2016/12/PI-AnnexinV.pdf.
  c. Incubate the cells in an orbital shaker at 37°C and 100 rpm for 20 min.
    **CRITICAL:** Cover the flask with aluminum foil to block light exposure. Work in a dimmed light environment for the remaining steps.
2. Rinsing of cells.
  a. Collect media with floating cells into a 50 mL conical tube.
  b. Spin down the cells in the media at 200 g for 10 min by using an Avanti J-E centrifuge with a JS-5.3 rotor.
  c. Keep the remaining adherent cells in the T75 flask in 5 mL of 1× PBS to rinse away the dye and prevent drying while the suspension is spinning.
  d. By using a light microscope, check that adherent cells are not lost.
  e. Resuspend the cell pellet into 20 mL fresh media (e.g., RPMI 1640) and transfer back into the original T75 cell culture flask after aspirating off the PBS.

***Note:*** Harsh rinsing of the adhered cells might cause loss. Gentle rinsing is recommended.

***Note:*** Resuspension can be done in media with or without supplement.

### Fixation, permeabilization, and DAPI counterstaining

**Timing: 1–2 h**

This step prepares cells for immunostaining and does not depend on the previous step for staining live cells.

3. Fixing the cells.
  a. Add ∼2.73 mL of fixation reagent to 20 mL of cells.
  b. Incubate the cells at 37°C with 100 rpm shaking in an orbital shaker for 15 min.
  c. Collect all cells by tapping on the flask or pipetting up and down to gently detach the adhered cells.

***Note:*** Have the T75 flask sit vertically for a least 1 min to allow all cells to accumulate in media at the bottom. Additional tapping and pipetting up and down can be done prior to the addition of the fixative at step (3a).

4. Permeabilizing cells while quenching aldehyde fixation.
  a. For each T75 flask, prepare a total of four 50 mL conical tubes.
  b. Fill each tube with 40 mL of permeabilizing solution at RT.
  c. Equally distribute the fixed cells between the four tubes with ∼5.7 mL/tube.
  d. Incubate the cells at 20°C–23°C on a rocking platform (VWR Model 100 at the setting 2) for 15 min.

***Note:*** During this incubation, Tris will react with formaldehyde, rendering it inactive. When 160 mL of permeabilization/quenching buffer is added to ∼23 mL of cells in 4% formaldehyde, the molar amount of Tris (0.032 moles) slightly exceeds that of formaldehyde (∼0.03 moles), causing fixation to be quenched. However, some additional cell fixation takes place during this step.

5. Two-step centrifugation and DAPI staining.
  a. Use a centrifuge to spin the four 50 mL tubes for 10 min at 200 *g* and 4°C.
  b. Aspirate most of the supernatant away to leave 2–3 mL in each tube.
  c. Resuspend the cell pellets into the remaining 2–3 mL of the supernatant.
  d. Combine the suspensions from all the 50 mL tubes into a single 15 mL conical tube (collecting about 10 mL of volume).
  e. Count the total number of cells for step j.
  f. Add DAPI solution to a final concentration of 5 μg/mL.
  g. Incubate at 20°C–23°C on a rocking platform at 2 rpm for 30 min.
  h. Centrifuge the cells at 200 g and 4°C for 10 min.
  i. Carefully aspirate away the supernatant from the cell pellet.
  j. Resuspend the cell pellet in 1× PBS to a final concentration of 1 × 10^6^/mL.

***Note:*** At this step, the resuspended cells can be stored at 4°C for a few days following addition of sodium azide (e.g. 0.02%).

### Staining with fluorescent probes and cell attachment to the glass

**Timing: ∼ 16 h**

6. Incubation with probes.
  a. Transfer 0.2 mL of the cell suspension (about 200,000 cells) into a 0.5 mL Eppendorf tube.
  b. Add ATTO-643 phalloidin conjugate and primary antibodies to the 0.2 mL cell suspension.
    ***Note:*** In this protocol, we use phalloidin at a final concentration 100 nM by a 1:100 dilution of a 10 μM stock solution. We immunostained the RPMI 8226 cells with TUBB4A antibodies at a final concentration of 10 μg /mL from 1:30 dilution of a 300 μg /mL stock concentration obtained following the probe conjugation with Biotium CF568 Mix-n-Stain kit according to the manufacturer’s instructions: https://biotium.com/wp-content/uploads/2017/10/PI-Mix-n-Stain-Antibody-Labeling-Kits.pdf.
  c. Cover the Eppendorf tubes with aluminum foil to prevent light exposure and incubate them horizontally ∼15 h on a rocking platform (VWR Model 100 at the setting 2) at 4°C.
    ***Note:*** Minimize exposure to light by performing all staining in a dimmed light environment.
    ***Alternatives****:* Staining can also be performed by incubating in an orbital shaker at 37°C and 100 rpm for 1–2 h.
7. Dilution of probes and attachment of cells to the glass.
  a. Place the 25 mm cover glass with the labeled side up at the bottom of a flat bottom 50 mL tube.
  b. Fill the tube with 50 mL of 1× PBS.
  c. Transfer the entire 0.2 mL of the stained cell suspension into the 50 mL tube.
    **CRITICAL:** Assure that the coated and labeled side of the cover glass is facing up. Otherwise, cap the tube, then shake and rotate it to position the cover glass correctly.
  d. Centrifuge the cells at 200 g and 4°C for 10 min; from this point, handle the tube with care to avoid cell detachment (problem 1).
  e. Store for up to 2 days at 4°C until imaging.
    ***Note:*** For longer storage of the stained cells at 4°C, add sodium azide at step (7e). The stained cells can be stored for up to 3 weeks.
    **CRITICAL:** A small amount of conjugated antibodies will attach to the poly-lysine at glass surface, staining it weakly (Video S1). This staining will increase background in the wide-field fluorescence imaging, but not in confocal microscopy.
    Video S1. 3D rendering of RPMI 8226 cells in a tile scan, related to step 4 Overview of cell density in a 63× objective tile scan of 6 fields wide and 5 fields high at 1.37 mm^2^. F-actin (phalloidin-ATTO643, gold) and DNA (DAPI, blue) staining are shown.

### Mounting and microscopy image acquisition and processing

**Timing: 1 day**

8. Mounting cover glass into a Chamlide magnetic chamber.
  a. Carefully decant PBS out of the tube to leave 5–10 mL.
  b. Un-stick the glass from the bottom of the tube with something long and sharp, such as an Abdos 3 mL low density polyethylene Pasteur pipette tip cut with scissors at a sharp angle.
  c. Using the cut Pasteur pipette, position the glass horizontally in the tilted tube, while still submerged in the liquid (Figure 1).

**Figure 1:**
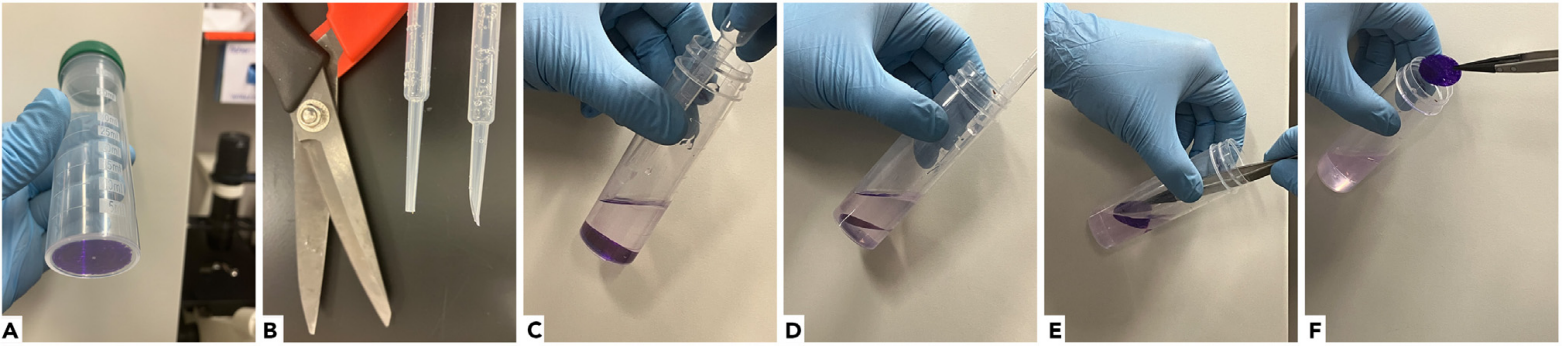
Stepwise demonstration of removal of a 25 mm cover glass from a flat bottom 50 mL tube (A) 25 mm cover glass stained purple for visualization purposes at the bottom of a tube. (B) Plastic transfer pipette tip before (center) and after (right) being cut with scissors (left) to create a tool for gentle detachment of the cover glass from the bottom of the tube. (C) After slow decanting of 40–45 mL of the liquid, the cut pipette is used to grab the edge of the glass. (D) Cover glass is repositioned by the cut pipette to make it accessible to the tweezers. (E) The tweezers gently engage the cover glass. (F) The cover glass is lifted out of the tube by the tweezers.
  d. Pick up the glass with forceps/tweezers (Figure 1, problem 2).
  e. Check that the labeled side (e.g., side with letter R) is facing up.
  f. Transfer the cover glass into a Chamlide magnetic chamber base (problem 3).
  g. Position the top part of the magnetic chamber on top of the cover glass, so that the magnet can press the o-ring against the glass, and gently rotate to ensure a proper seal.
  h. Slowly add 1 mL of PBS to the edge of the glass; strong flow can detach cells.
    ***Note:*** If high quality bright-field imaging is required, the chamber should be slightly over-filled with PBS, which requires about 4 mL of PBS. After the glass lid is placed on top of the over-field chamber, there will be no air gap under the lid, and no meniscus to refract trans-illumination light.
    ***Note:*** Skip step (8i) if a high numerical aperture water immersion lens is available.
  i. If imaging with an oil lens, carefully aspirate PBS and slowly add mounting medium in an amount sufficient to cover the cells.
  j. Place the lid on the Chamlide chamber.
9. Microscopy and image processing.
  a. Acquire images with a confocal microscope, such as a Leica DMi8 microscope equipped with a Yokogawa CSU-W1 Spinning Disk and an Andor Zyla 4.2 sCMOS camera controlled by Andor Fusion software (problem 4).
    ***Note:*** The imaging parameters used were as follows: samples were mounted in PBS on a 63× NA1.2 water immersion lens. Images were acquired using a 107 nm pixel size, 200 nm Z step, 22 μm Z range, with 30 ms exposure time, and 100% power for 640 nm, 561 nm, 488 nm and 405 nm lasers, used with emission filters 700/75 nm, 600/50 nm, 525/50 nm, and 450/50 nm. The Spinning Disk pinholes size was 50 μm.
    ***Note:*** A 63× NA1.4 oil lens and 63× NA1.2 water lens provide comparable image quality, with the water lens providing an advantage of refractive index matching, eliminating depth-dependent spherical aberration. The signal to noise of dye-probe combinations listed in this protocol is very high, and photobleaching is not a problem even without antifade, so that imaging with a water lens in PBS is preferable.
    ***Alternatives:*** Any confocal microscope can be used for imaging.
  b. Deconvolve the images with Andor Fusion software, or another deconvolution software (problem 5).
  c. Process images with ImageJ^8^ and Imaris software.

## Expected outcomes

**Figure 2:**
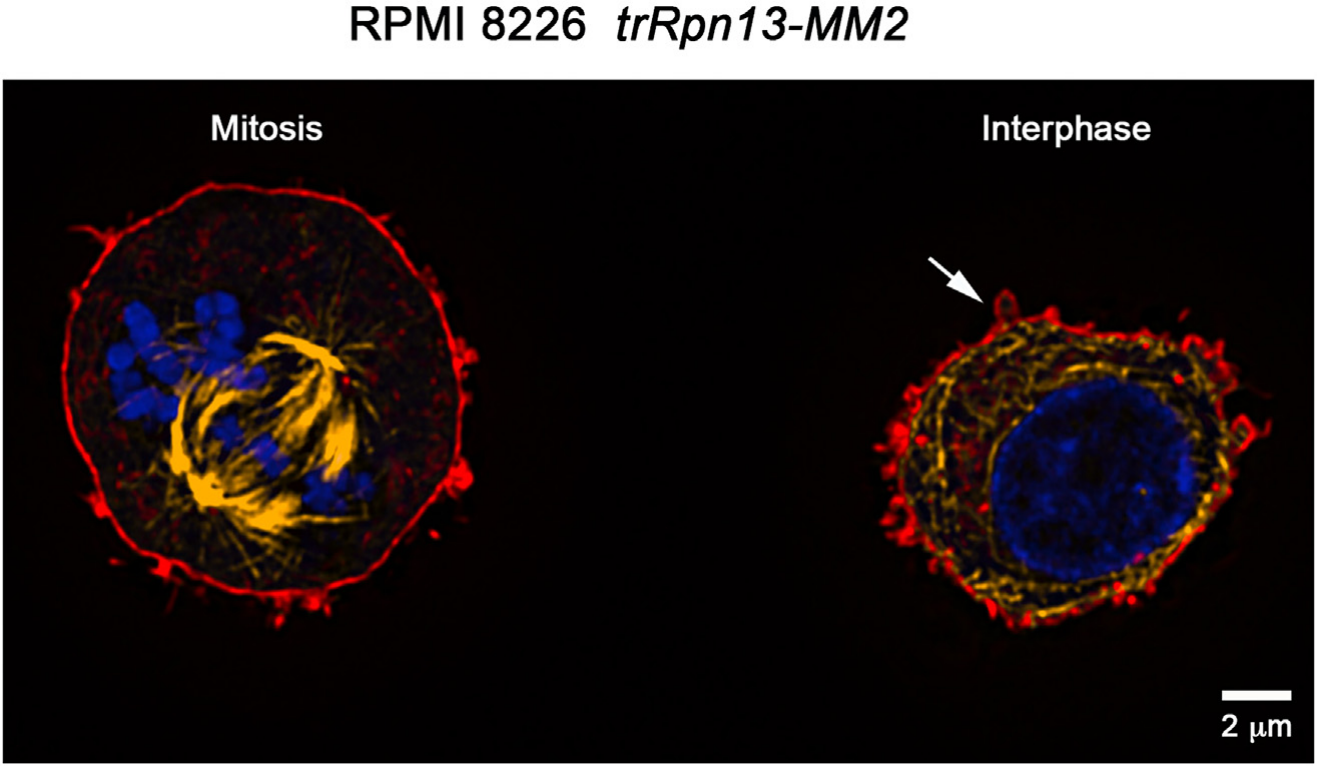
Immunofluorescence staining of RPMI 8226 *trRpn13-MM2* cells Z-slices from a confocal microscopy image of a sample prepared following the described method showing RPMI 8226 *trRpn13-MM2* cells undergoing mitosis (left) and at interphase (right) with fluorescence staining for F-actin (phalloidin-ATTO643, red), β-tubulin (TUBB4A-CF568, yellow) and DNA (DAPI, blue). Membrane blebbing is indicated by a white arrow. Scale bar is 2 μm.

This protocol was designed for high-resolution microscopy of non-adherent cells to minimize distortion of cell morphology. With these steps, we were able to achieve reasonable cell density across a 25 mm diameter glass cover slide (as illustrated in Video S1), which was sufficient to measure statistically significant changes across different cell lines. Unperturbed cell features, including morphology of small protrusions and blebs on the cell surface, allows observation of minute changes in the organization of the cytoskeleton, especially when combined with 3D visualization software, such as Imaris (Figure 2; Videos S2 and S3). This method should be applicable not only for cytoskeleton labeling, but to image of any cellular targets that can be labeled following typical formaldehyde fixation and detergent permeabilization. The main advantage of this method is avoiding even partial flattening of cells on the cover glass, preserving overall morphology of suspension cells, and fragile structures such as blebs. Such preservation of morphology should be beneficial for automated analysis with imaging tools such as Imaris and FIJI/ImageJ, which was not part of this study.^1^

Video S2. 3D rendering of two RPMI 8226 cells at interphase on a cover glass, related to step 4 Cells are labeled with actin stain (phalloidin, red), β-tubulin antibody (green) and DNA stain (blue). Both cells have a spherical shape. In these cells, tubulin staining highlights the outside boundary of the cytoplasm, as microtubules are positioned along plasma membrane. Actin staining highlights hair-like structures of these cells. As illustrated at the zoomed-in part of this video, one of the cells is only attached to the glass through actin-rich protrusions, with a substantial gap between the microtubule-delineated cytoplasm and glass surface. The cover glass is stained by labeled antibody (presented in green).

Video S3. 3D rendering of an RPMI 8226 mitotic cell on a cover glass, related to step 4 Cells are stained with DAPI (blue), anti- β-tubulin antibodies (green) and phalloidin conjugate (red), which demonstrates substantial blebbing on the surface. No apparent artifacts caused by attachment to the glass surface are visible.

## Limitations

Firstly, this protocol was tested only on one type of semi-adherent cell line, RPMI 8226. Different cell types may require fine-tuning of the protocol. Additionally, we did not include a blocking step, since it was not required for the specific antibodies used for our study. If blocking is required, the cell pellet can be re-suspended into the blocking buffer instead of PBS at step 5j.

## Troubleshooting

### Problem 1

Cell detachment from the cover glass at Step 7d.

### Potential solution

• Check that the coated side of the cover glass is facing up.
• Increase the g-force of centrifugation staying below 1000 g when spinning the cells down to attach them to the cover glass.
• Increase the concentration of poly-lysine up to 0.1% when treating the cover glasses.
• Try using Cell-Tak (Corning at 3.5 μg/cm^2^) as an alternative to Poly-L coating, according to the manufacturer’s instructions: https://www.scientificlabs.co.uk/handlers/libraryFiles.ashx?filename=Manuals_3_354240_A.pdf.

***Note:*** Floating or loosely attached cells do not interfere with the analyses as they are readily distinguished in a Z stack.

### Problem 2

Cover glass is cracked or scratched during handling at Step 8.

### Potential solution

• To reduce risk of cover glass cracking, use Carbon-Fiber Tip Tweezers.

### Problem 3

Cells lost from the cover glass during mounting at Step 8.

### Potential solution

• Check that the coated side of the cover glass is facing up at Step 8e.

### Problem 4

Photobleaching is encountered at Step 9.

### Potential solution

• Use antifade mounting media.
• For cyanine dyes, try Biotium Drop-n-Stain EverBrite Mounting Medium.

### Problem 5

Cells that are supposed to appear spherical are elongated in appearance.

### Potential solution

• Use mounting media with a refraction index that matches the objective immersion media, such as PBS for water immersion lenses or oil refraction index matched mounting media for oil lenses, for example SlowFade Glass.
• Check deconvolution software settings for refractive index settings of mounting and immersion media, as well as the set distance in the glass settings. Most deconvolution software is designed to correct for spherical aberration, but if software settings are incorrect aberration will be introduced.

## Resource availability

### Lead contact

Further information and requests for resources and reagents should be directed to and will be fulfilled by the lead contact, Kylie Walters (kylie.walters@nih.gov).

### Technical contact

Technical questions on executing this protocol should be directed to and will be answered by the technical contacts, Vasty Osei Amponsa (vasty.oseiamponsa@nih.gov) and Valentin Magidson (valentin.magidson@nih.gov).

### Materials availability

All materials used in this protocol are commercially available in the United States and their information is provided in the key resources table.

### Data and code availability

This study did not generate/analyze datasets or code.
